# RibDif2: expanding amplicon analysis to full genomes

**DOI:** 10.1093/bioadv/vbad111

**Published:** 2023-08-21

**Authors:** Robert Murphy, Mikael Lenz Strube

**Affiliations:** Section for Ecology and Evolution, Department of Biology, University of Copenhagen, Copenhagen 2100, Denmark; Department of Biotechnology and Biomedicine, Technical University of Denmark, Kongens Lyngby 2800, Denmark; Department of Biotechnology and Biomedicine, Technical University of Denmark, Kongens Lyngby 2800, Denmark

## Abstract

**Motivation:**

As previously described, amplicon analysis of the bacterial 16S gene has several limitations owing to fundamental characteristics of both the 16S gene and technological restrictions. Previously, RibDif was introduced to help quantify these limitations by detailed analysis of a given genera and the 16S gene profile of its members, notably multiplicity and divergence of 16S alleles within genomes as well as shared alleles between species. Apart from using amplicon analysis for only the 16S gene, amplicons derived from genus-specific genes or even functional genes are increasingly being utilized. Moreover, long-read technologies are progressively being used to sequence longer amplicons, and since these inherently contain more information, they may likely alleviate the issues proposed in RibDif.

**Results:**

Taking these phenomena into account, we here propose RibDif2. RibDif2 retains the 16S-optimized functionality of the original RibDif but can now run any set of primers on any part of the genome in any set of organisms, be it prokaryote, eukaryote, or archaea. We demonstrate this new functionality by showing full species resolution of *Pseudoalteromonas* using complete rRNA-operon amplicons, as well as selection of optimally discriminatory primers for *Staphylococcus* and *Pseudomonas*. Moreover, we show a potential bias toward terrestrial bacteria relative to marine ones for primers amplifying biosynthetic gene clusters and lastly suggest optimal primers to differentiate the members of the insect genus *Drosophila*. We believe that RibDif2 will facilitate the work of all scientists using amplicon sequencing, especially in the era of long-read sequencing.

**Availability and implementation:**

Ribdif2 is freely available at https://github.com/Rob-murphys/ribdif.

## 1 Introduction

Amplicon-based sequence analysis is a low-cost and high-throughput method traditionally used to profile the microbial composition of an environment (Gupta *et al.* 2019, Bharti and Grimm 2021) or ecological niche. These collections of microbes, or microbiomes, are now recognized as key players for the health, disease, and performance of most niches ranging from humans (Hou *et al.* 2022) to plants (Trivedi *et al.* 2020). Given the importance of accurately profiling the composition of such microbiomes, methods for estimation of their composition have received substantial attention, such as through culturing (Gupta *et al.* 2019) or metagenomic sequencing (Rausch *et al.* 2019).

High-throughput amplicon sequencing is conceptually driven by a PCR reaction targeting conserved regions flanking highly variable ones, followed by sequencing of the resulting amplicons, which, ideally, contain sufficient variance for analytic inference. A common target is the 16S rRNA gene, ubiquitous in bacteria and archaea, since this gene has a uniquely alternating pattern of high conservation and high variation (Větrovský and Baldrian 2013). Amplification of the V3-V4 region spanning ∼450 bp in particular has become standard (Bharti and Grimm 2021). Although the 16S rRNA gene is by far the most widely used target for amplicon sequencing, multiple other genes have been targeted not only for alternative taxonomy but also in investigations of secondary metabolite potential as well (Geers *et al.* 2022). Common for most of these analyses is the use of short-read sequencing, notably delivered by the paired-end Illumina technology which, despite providing high accuracy, does not allow sequencing of amplicons longer than ∼500 bp. Recently, however, the maturation of the Nanopore and PacBio technologies now allows for routine sequencing of much longer DNA, including long amplicons.

Although amplicon sequencing is routinely used, the method has several fundamental limitations. In particular, the widely adopted use of the 16S rRNA gene comes with multiple rarely considered limitations as previously described (Větrovský and Baldrian 2013, Johnson *et al.* 2019) and addressed in our previous paper (Strube 2021). As a short re-iteration of these limitations, we mainly highlight that a genome can have, and often has, multiple and different alleles of the 16S rRNA gene (Větrovský and Baldrian 2013), and that these alleles may moreover be shared across several different species (Johnson *et al.* 2019). The first issue leads to artificial inflation of observed diversity along with potential deflation of abundance, since amplicons derived from such a genome will result in several different ASVs rather than one. The second issue prohibits accurate species differentiation, since such an amplicon cannot uniquely be assigned to a single species. Although the introduction of the amplicon sequence variant (ASV) has been revolutionary for the field of microbial ecology (Callahan *et al.* 2017), the issues described above are likely exacerbated by its use. Prior to the introduction of the ASV, amplicons were routinely clustered (e.g. at 97% similarity) into operational taxonomic units (OTUs), which initially was motivated by technical limitations, but had the inherent benefit of averaging out the biological (and technical) variation giving rise to the issues described above. However, the use of the OTU evidently comes with a greatly diminished level of resolution making the ASV the superior methodology as long as its limitations are kept in mind.

Previously, we have addressed these issues with RibDif (Strube 2021), which was written to quantitatively highlight potential issues arising from the use of 16S rRNA gene amplicons analysis. Although RibDif has been widely used to investigate which genera can be meaningfully described by 16S sequencing and—equally important—which genera cannot, the software was restricted to the 16S rRNA gene (Strube 2021). Perhaps equally important, RibDif would routinely demonstrate how full-length 16S amplicons (e.g. V1–V9) could resolve species when V3–V4 amplicons of the same gene could not. Moreover, we noted an increase in the use of non-16S primers, both for taxonomic discrimination and functional profiling. As an accommodation of the developments in the field, namely (i) the profiling of specific genera in complex samples by specialized non-16S primers (Strube *et al.* 2018, Lauritsen *et al.* 2021), (ii) verification of primers targeting functional domains (Lauritsen *et al.* 2021, Geers *et al.* 2022), or (iii) the use of novel technologies for long-range amplicons, we decided to develop RibDif2.

RibDif2 retains the exact functionality of RibDif, namely the analysis of the 16S gene, but has been vastly extended to accommodate the challenges described above. Apart from the special case of the 16S gene, RibDif2 will now readily provide the amplification rate and resolving power of any set of primers on any set of genomes, including Eukaryotes and Archaea. Moreover, visual feedback has been improved by inclusion of association networks to summarize genomic overlap for a given primer set. Furthermore, we completely rewrote the tool in Python and have made it available for users less familiar with the command line by offering it as a web server at biolib.com/ATGATAGGCGACCGGACC/RibDif2/.

## 2 Methods

**Figure 1.**
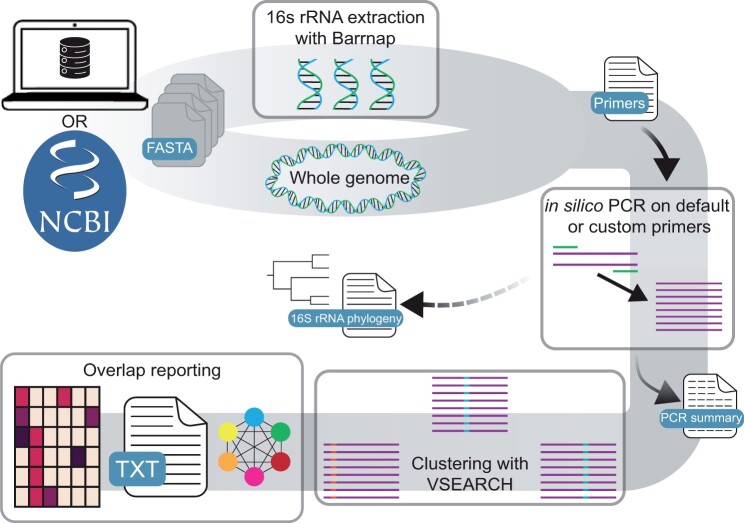
Schematic overview of the RibDif2 workflow, with optional (dashed lines) or mandatory (solid lines) outputs. In brief, the user initiates the analysis by either specifying a set of local genomes or alternatively a specific genus. In the latter case, all high-quality and complete genomes are downloaded from NCBI. In either case, the user has the choice of specific analysis of the 16S rRNA gene or the complete genome. Amplicons are then *in silico* PCR amplified and resultant amplicons clustered. Finally, an overlap report is generated with various graphical and text-based outputs.

Ribdif2 is freely available at https://github.com/Rob-murphys/ribdif (Fig. 1). Similar to version 1, RibDif2 only requires a genus for a standard run, in which case it will evaluate the allelic multiplicity and species overlap when using V3–V4 and V1–V9 16S rRNA primers [see the original paper (Strube 2021) for details].

Alternatively, and as the key novelty, RibDif2 can now run on whole genomes by specifying the -w/–whole-genome argument which skips rRNA prediction and instead searches the whole genome directly for the desired amplicon. Following successful *in silico* amplification, VSEARCH (Rognes *et al.* 2016) clusters amplicons at a default identity of 100%, although this can be specified by the user (by specifying the -i/–id argument as a value between 0.7 and 1.0). Membership to these clusters is processed and summary graphics of allele multiplicity and species overlap are provided, with the latter also being presented as text along with presence/absence heatmaps and networks for improved visual clarity. Furthermore, users wishing to analyze local collections of genomes for IPR reasons can do so, although this mode of analysis disregards taxonomic inference and may be affected by contamination and incompleteness. Both additions allow RibDif2 to now work on any domain of life provided complete enough genomes exist. As a default, RibDif2 only downloads and analyzes complete genomes from NCBI, since fragmented and/or metagenomic-derived genomes are certain to have fragmented rRNA operons and/or high levels of contamination. Use of fragmented genomes can be enabled by specifying the -f/–frag argument. In case the user is targeting a non-bacterial genus, RibDif2 will automatically search for genomes at chromosome-level completion since these are rarely complete.

RibDif2 is fully implemented in python allowing for simple installation through pip. Using default settings, RibDif2 will download and analyze all 2635 genomes of the *Escherichia* genus in 50 and 60 min on standard and whole-genome mode, respectively, of which rRNA prediction and *in sillico* PCR are still the major bottlenecks on 10× dual-core Intel(R) Xeon(R) W-2155 CPU @ 3.30 GHz. However, analysis of smaller genera is accomplished in a much speedier manner.

For testing in whole-genome mode, we used full-length rRNA-operon primers (Seol *et al.* 2022), *tuf-*gene primers (Strube *et al.* 2018, Ahle *et al.* 2022), *rpoD-*gene primers (Lauritsen *et al.* 2021), primers targeting the adenylation domain of NRPS biosynthetic gene clusters (Geers *et al.* 2022), and lastly, primers targeting the eukaryotic 12S and COI genes (Marquina *et al.* 2019) (see Supplementary Table S1 for full details).

## 3 Results

We present RibDif2, an extensive overhaul of the popular RibDif software. We demonstrate the novel features of RibDif2 with four common use-cases: (i) validation of long-range rRNA amplicons for species resolution, (ii) high-throughput testing of genera-specific primers in minutes rather than weeks, (iii) optimal choice of primers targeting biosynthetic gene clusters depending on ecological niche, and lastly (iv) investigation of non-bacterial targets, such as primers for invertebrate, archaeal and mammalian genomes.

**Figure 2.**
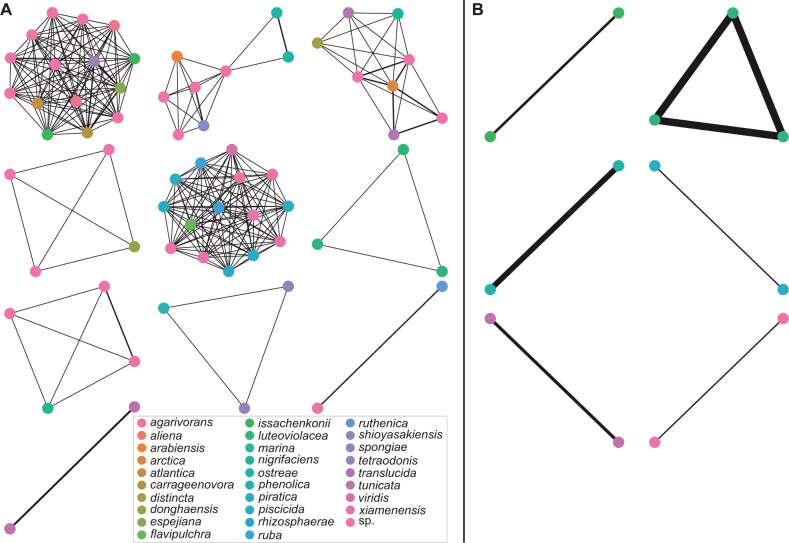
Network visualization of genome overlap as provided by RibDif2. Here, genomes are represented as nodes, which are connected by edges if nodes/genomes have overlapping alleles. Non-connected nodes/genomes are excluded, and edge width is scaled by the number of shared alleles. Across 68 genomes of *Pseudoalteromonas*, an extensive overlap is observed between amplicons derived from (A) V3–V4 primers in contrast to amplicons derived from (B) V1–V9 primers. Of note, complete rRNA-operon amplicons have no overlap in this genus.

First, we demonstrate the improved capacity of RibDif2 by extending the analysis of the original software, i.e. by using standard V3V4 and V1V9 primers on the genus *Pseudoalteromonas (*Strube 2021), a genus difficult to separate into species by 16S rRNA sequencing (Strube 2021). Again, this genus is poorly resolved with both V3V4 and V1V9 primers, evident by these sets of primers producing amplicons which overlap in 75.0% and 42.9% species, respectively. RibDif2, however, allows analysis across the entire genome and by using this whole-genome mode, we test primers spanning the complete rRNA operon (e.g. across the 16S-ITS-23S genes). In contrast to the V3–V4 and V1–V9 primers, these primers produce amplicons completely resolving individual members of *Pseudoalteromonas* and can thus be used for species resolution (Fig. 2), provided that one uses a sequencing technology appropriate for these longer sequences.

Second, we simulate a user who has correctly realized that the 16S rRNA gene cannot separate species within *Staphylococcus* nor *Pseudomonas* and is instead investigating sets of alternative primers targeting the *tuf* and *rpoD* genes, respectively, both of which are single copy. Here, the analysis shows the *tuf* primers of Ahle *et al.* (2022) to not only cover 99.5% of genomes within the *Staphylococcus* genus but also that the resulting amplicons are completely specific to each species, i.e. have no amplicon overlap. Similarly, the *rpoD* primers of Lauritsen *et al.* (2021) match 99.6% of all *Pseudomonas* genomes and produce amplicons specific for each species, hence making both these primers well suited for investigating their respective genera.

Third, amplicon sequencing is routinely used for profiling the richness of biosynthetic gene cluster diversity, often by use of primers targeting non-ribosomal peptide synthetases or polyketide synthetases (Geers *et al.* 2022). These primers are often heavily degenerate and since they are usually designed with specific bacteria or environments in mind, they may not be optimal for other ecological niches. Here, we used 8 primer sets targeting these domains (Table 1 and Supplementary Table S1) on representatives of both marine (*Pseudoalteromonas*) and terrestrial (*Nocardia*) bacteria and observed a clear bias toward the terrestrial genus: *Nocardia* genomes were amplified by 7/8 primers resulting in an average genome-wise amplification rate of 81%, but conversely, *Pseudoalteromonas* genomes where only matched by 5/8 primers, for which the average amplification rate was only 17% (Table 1). Although the scope of this analysis is insufficient for concluding a terrestrial-vs-marine bias, the analysis does suggest that one should investigate multiple primers before applying them to a given environment. In this particular case, the results suggest that a marine system rich in *Pseudoalteromonas* should probably be analyzed by use of the MDPQQR primers for PKS and degNRPS primers for NRPS.

**Table 1.** Amplification rate and Shannon diversity of Non-Ribosomal Peptide Synthetase (NRPS) and Polyketide Synthase (PKS) targeting primers for Norcadia (terrestrial) and Pseudoalteromonas (marine). Shannon diversity is displayed as the sum of Shannon diversity across each base pair of the alignment.

Primer	Target	Norcadia		Pseudoalteromonas	
		Amplification rate	Shannon diversity	Amplification rate	Shannon diversity
degNRPS-4F/degNRPS-1R	NRPS	1	121.76	0.377	262.88
MTF2/MTFR	NRPS	1	100.48	0.377	206.61
A3F/A7R	NRPS	1	70.64	0	0
F/R	NRPS	0	0	0	0
degKS2F/degKS2R	KS	1	19.95	0.058	16.73
KSDPQQF/KSHGTGR	KS	0.667	13.85	0.072	26.33
KSLF/KSLR	KS	0.806	11.38	0	0
MDPQQRf/HGTGTr	KS	1	221.11	0.391	169.49

Lastly, RibDif2 can now analyze any domain of life, including eukaryotes. We demonstrate this by investigating the 19 available chromosome-level genomes of the *Drosophila* genus with insect specific *COI* primers and 12S primers (Supplementary Table S1). These two primers successfully amplify 13 and 12 genomes, respectively, suggesting *COI* primers to have some advantage, although neither primer set results in amplicons specific enough to resolve this genus completely, since *Drosophila subpulchrella* and *Drosophila suzukii* share alleles with both primer sets.

## 4 Discussion and conclusion

Although 16S rRNA gene amplicon sequencing as a technique has been fundamentally successful (Větrovský and Baldrian 2013, Johnson *et al.* 2019), several aspects inherent in biology limits its use. To reiterate, multiple alleles of the 16S gene are often found within each bacterial genome and some of these alleles may furthermore be found in the genomes of other species. As is inevitable in metataxonomic analysis of complex microbiomes, intragenomic allelic multiplicity will result in overinflation of α-diversity, whilst interspecies sharing of alleles will hinder species differentiation. The implications of these issues are not novel to this paper and have previously been raised by a number of publications (Větrovský and Baldrian 2013, Johnson *et al.* 2019). Accordingly, the software described in our previous paper (Strube 2021) sought to illuminate the scale of these limitations by providing users with quantitative estimations of the usefulness of 16S rRNA amplicon analysis for whichever genus was in focus.

Apart from merely using amplicons to analyze the 16S gene, however, we have observed an increased usage of non-16S amplicons for novel taxonomic and functional purposes and, along with the maturation of long-read technology, motivated us to introduce RibDif2. RibDif2 can now profile amplicons from any genomic region in any set of genomes and is hence useful for all scientists using amplicon sequencing.

We demonstrate the superiority of long, full rRNA-operon amplicons for resolving species within genera infamously difficult to profile with standard V3–V4 sequencing, suggesting that future metataxonomic analyses could benefit from the inclusion of long-read technologies. Moreover, we provide use-cases for scientists who wish to easily pick the correct primers for future experiments, be it detailed taxonomic analysis or functional profiling. Lastly, we demonstrate the expanded capacity of RibDif2 by concluding that primers targeting neither the COI or 12S regions can separate members of the higher eukaryote, *Drosophila*, into species.

In conclusion, we believe that RibDif2 is an invaluable tool for any researcher working with amplicon analysis. Along with the capacity to analyze which genera can be meaningfully separated into species by standard metataxonomic analysis, RibDif2 now allows easy and efficient selection of primers for any target, in any taxonomy and with any sequencing technology.

## Supplementary Material

vbad111_Supplementary_Data
